# Supplementary material for: Capturing additional genetic risk from family history for improved polygenic risk prediction
Source: Commun Biol. 2022 Jun 16;5:595. doi: 10.1038/s42003-022-03532-4 (PMC9203758; doi:10.1038/s42003-022-03532-4)
Supplement: Supplementary file 3 — Description of Additional Supplementary Files [file 42003_2022_3532_MOESM3_ESM.pdf]

Description of Additional Supplementary Files

File Name: Supplementary Data 1
Description: The source data underlying Figure 2.

File Name: Supplementary Data 2
Description: The source data underlying Figure 3d.
