# Supplementary material for: Capturing additional genetic risk from family history for improved polygenic risk prediction
Source: Commun Biol. 2022 Jun 16;5:595. doi: 10.1038/s42003-022-03532-4 (PMC9203758; doi:10.1038/s42003-022-03532-4)
Supplement: Supplementary file 6 — Reporting Summary [file 42003_2022_3532_MOESM6_ESM.pdf]

nature portfolio

Corresponding author(s): Celia Greenwood

Last updated by author(s): Feb 1, 2022

Reporting Summary

Nature Portfolio wishes to improve the reproducibility of the work that we publish. This form provides structure for consistency and transparency in reporting. For further information on Nature Portfolio policies, see our Editorial Policies and the Editorial Policy Checklist.

Statistics

For all statistical analyses, confirm that the following items are present in the figure legend, table legend, main text, or Methods section.

n/a	Confirmed	
☐	☒	The exact sample size (n) for each experimental group/condition, given as a discrete number and unit of measurement
☐	☒	A statement on whether measurements were taken from distinct samples or whether the same sample was measured repeatedly
☐	☒	The statistical test(s) used AND whether they are one- or two-sided<br>*Only common tests should be described solely by name; describe more complex techniques in the Methods section.*
☐	☒	A description of all covariates tested
☐	☒	A description of any assumptions or corrections, such as tests of normality and adjustment for multiple comparisons
☒	☐	A full description of the statistical parameters including central tendency (e.g. means) or other basic estimates (e.g. regression coefficient) AND variation (e.g. standard deviation) or associated estimates of uncertainty (e.g. confidence intervals)
☒	☐	For null hypothesis testing, the test statistic (e.g. F, t, r) with confidence intervals, effect sizes, degrees of freedom and P value noted<br>*Give P values as exact values whenever suitable.*
☐	☒	For Bayesian analysis, information on the choice of priors and Markov chain Monte Carlo settings
☒	☐	For hierarchical and complex designs, identification of the appropriate level for tests and full reporting of outcomes
☐	☒	Estimates of effect sizes (e.g. Cohen's d, Pearson's r), indicating how they were calculated

Our web collection on statistics for biologists contains articles on many of the points above.

Software and code

Policy information about availability of computer code

Data collection	No software was used for data collection.
Data analysis	Data analysis was performed using R version 4.0.5.

For manuscripts utilizing custom algorithms or software that are central to the research but not yet described in published literature, software must be made available to editors and reviewers. We strongly encourage code deposition in a community repository (e.g. GitHub). See the Nature Portfolio guidelines for submitting code & software for further information.

Data

Policy information about availability of data

All manuscripts must include a data availability statement. This statement should provide the following information, where applicable:

- Accession codes, unique identifiers, or web links for publicly available datasets
- A description of any restrictions on data availability
- For clinical datasets or third party data, please ensure that the statement adheres to our policy

Individual genotype and pheontype data from the UK Biobank (https://www.ukbiobank.ac.uk/) and the ALSPAC (http://www.bristol.ac.uk/alspac/) are available through successful applications to the research committees. The ALSPAC website contains details of all the data that is available through a fully searchable data dictionary and variable search tool (http://www.bristol.ac.uk/alspac/researchers/our-data/). A computational toolkit implementing the latent factor model developed in this study is available at https://github.com/tianyuan-lu/PRS-FH-Prediction.

Field-specific reporting

Please select the one below that is the best fit for your research. If you are not sure, read the appropriate sections before making your selection.

☒ Life sciences ☐ Behavioural & social sciences ☐ Ecological, evolutionary & environmental sciences

For a reference copy of the document with all sections, see nature.com/documents/nr-reporting-summary-flat.pdf

Life sciences study design

All studies must disclose on these points even when the disclosure is negative.

Sample size	No sample size calculation was performed. This study leveraged the UK Biobank and the ALSPAC cohort, two of the largest genotyped cohorts to ensure sufficient statistical power.
Data exclusions	This study excluded individuals of non-European ancestries. This is because polygenic risk scores developed using European ancestry populations are known to have attenuated predictive performance when applied to other populations.
Replication	The main purpose of this study is to propose a computational framework. Effectiveness of this computational framework was validated using the UK Biobank and the ALSPAC cohort. No additional data were used for replication.
Randomization	Randomization was not performed. Age, sex and other population characteristics that may confound genetic associations have been included as covariates in linear regression models.
Blinding	The UK Biobank and the ALSPAC cohorts were established to represent the general population where participants volunteered to participate, thus blinding was not relevant to this study.

Reporting for specific materials, systems and methods

We require information from authors about some types of materials, experimental systems and methods used in many studies. Here, indicate whether each material, system or method listed is relevant to your study. If you are not sure if a list item applies to your research, read the appropriate section before selecting a response.

Materials & experimental systems

n/a	Involved in the study	
☒	☐	Antibodies
☒	☐	Eukaryotic cell lines
☒	☐	Palaeontology and archaeology
☒	☐	Animals and other organisms
☐	☒	Human research participants
☒	☐	Clinical data
☒	☐	Dual use research of concern

Methods

n/a	Involved in the study	
☒	☐	ChIP-seq
☒	☐	Flow cytometry
☒	☐	MRI-based neuroimaging

Human research participants

Policy information about studies involving human research participants

Population characteristics	Population characteristics are summarized in Supplementary Tables. The UK Biobank performed genome-wide genotyping using Affymetrix arrays based on DNA extracted from blood samples provided by the participants. The genotypes were imputed to the Haplotype Reference Consortium reference panel. Deep phenotyping of the UK Biobank participants was conducted upon the initial assessment visit, including a wide variety of anthropometric measurements, blood and urine biomarkers, etc. Genotyping of the children initially recruited into the ALSPAC cohort was conducted using the Illumina HumanHap550 quad genotyping platforms. The genotypes were imputed to the 1000 Genomes Phase 3 reference panel.
Recruitment	Between 2006-2010, the UK Biobank recruited and genotyped approximately 500,000 middle aged and older participants at multiple assessment centers located in the United Kingdom. Participants in the UK Biobank were slightly healthier, less obese, and less likely to smoke and consume alcohol compared to the general population. However, these participation biases are not directly relevant to this study. From 1991 to 1992, the ALSPAC cohort recruited 14,541 pregnancies in the Bristol and Avon areas in the United Kingdom. In addition, 913 pregnancies were enrolled in later phases of the study. The total sample size for analyses using data collected after the age of seven is therefore 15,454 pregnancies, resulting in 15,589 foetuses. Of these, 14,901 were alive at 1 year of age.
Ethics oversight	Ethical approval for the study was obtained from the ALSPAC Ethics and Law Committee and the Local Research Ethics Committees and the UK Biobank Access Sub Committee..

Note that full information on the approval of the study protocol must also be provided in the manuscript.
